# Machine Learning-Aided Inverse Design and Discovery of Novel Polymeric Materials for Membrane Separation

**DOI:** 10.1021/acs.est.4c08298

**Published:** 2024-12-16

**Authors:** Raghav Dangayach, Nohyeong Jeong, Elif Demirel, Nigmet Uzal, Victor Fung, Yongsheng Chen

**Affiliations:** † School of Civil & Environmental Engineering, Georgia Institute of Technology, Atlanta, Georgia 30332, United States; ‡ Department of Civil Engineering, Abdullah Gul University, 38039 Kayseri, Turkey; § School of Computational Science and Engineering, Georgia Institute of Technology, Atlanta, Georgia 30332, United States

**Keywords:** machine learning, polymeric membrane, separation, inverse design, material discovery

## Abstract

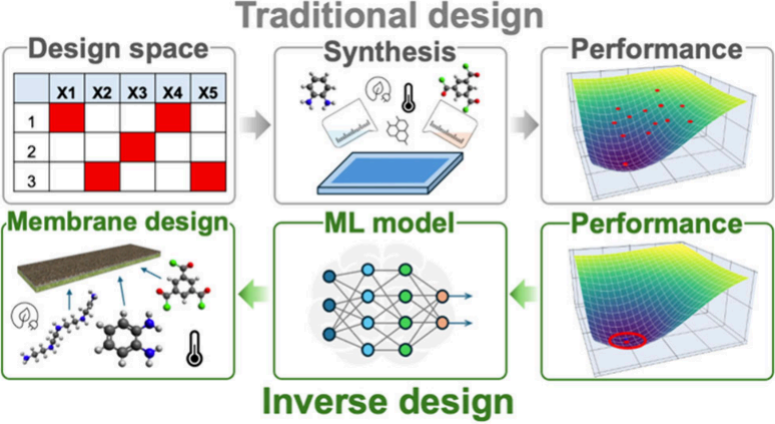

Polymeric membranes have been widely used for liquid and gas separation in various industrial applications over the past few decades because of their exceptional versatility and high tunability. Traditional trial-and-error methods for material synthesis are inadequate to meet the growing demands for high-performance membranes. Machine learning (ML) has demonstrated huge potential to accelerate design and discovery of membrane materials. In this review, we cover strengths and weaknesses of the traditional methods, followed by a discussion on the emergence of ML for developing advanced polymeric membranes. We describe methodologies for data collection, data preparation, the commonly used ML models, and the explainable artificial intelligence (XAI) tools implemented in membrane research. Furthermore, we explain the experimental and computational validation steps to verify the results provided by these ML models. Subsequently, we showcase successful case studies of polymeric membranes and emphasize inverse design methodology within a ML-driven structured framework. Finally, we conclude by highlighting the recent progress, challenges, and future research directions to advance ML research for next generation polymeric membranes. With this review, we aim to provide a comprehensive guideline to researchers, scientists, and engineers assisting in the implementation of ML to membrane research and to accelerate the membrane design and material discovery process.

## 1 Introduction

The precise separation of nanoscale molecules and ions from diverse solutions has gained significant importance in various industries over the past few decades.^1^ Membrane technology has emerged as an effective strategy to achieve this goal due to its high separation and energy efficiency, low capital cost, and easy scalability. The benefits associated with membrane technology has led to its utilization for a variety of applications such as wastewater treatment, water purification, gas separation, and resource recovery.^2,3^ Polymeric materials are at the forefront of membrane manufacturing as a result of their outstanding processability, high versatility, as well as exceptional mechanical and chemical stability.^4^ These polymers possess distinctive chemical and physical characteristics, which can be tailored to form multifunctional membranes.^5^ This diversity allows engineers and scientists to fine-tune polymeric membranes according to their individual needs such as high permeability and selectivity.

With the growing use of polymeric membranes for different applications, a permeability-selectivity trade-off has been observed due to their intrinsic limitations.^6−8^ This implies that polymeric membranes with high permeability typically possess low selectivity and vice versa. Investigating an exact property-process-structure relationship to balance this trade-off is a complex task due to three primary factors: the presence of numerous features such as material properties and synthesis variables, the vast material design space, and the lack of complete understanding of the physics and chemistry of sophisticated material systems.^9,10^ Traditional membrane fabrication is based on a “bottom-up” approach wherein the polymeric membranes are selected and put through an iterative trial-and-error process of adaptation and testing to improve membrane performance metrics.^11^ These approaches for membrane design are associated with being laborious and resource intensive. Alternatively, computational simulation tools such as molecular dynamics simulation (MD) and density functional theory (DFT) have shown good potential in the prediction of material structures and performance to varying degrees.^12,13^ However, it is important to note that these models require high computational demands and thus their applications are often limited to simpler conditions.^14^

Instead of using a “bottom-up” approach for membrane design, where material properties and performance metrics are calculated after membrane synthesis, scientists need to revamp their design approach to an inverse design methodology.^15^ This approach facilitates effective exploration of the membrane design space, enabling the identification of novel membrane materials and optimal fabrication conditions to achieve desired objectives.^16^ In this context, artificial intelligence (AI) has facilitated groundbreaking advancements in the field of design and discovery of membrane materials.^17−19^ AI refers to the study of computer programs or systems that can mimic human cognitive functions in data analysis, decision-making, and problem-solving in order to accomplish tasks, such as understanding natural language, learning from experience, and adapting to new situations.^20^ Machine learning (ML) is a branch of AI focused on the development of algorithms that leverage data to make predictions and decisions.^21^ ML has emerged as a viable alternative to conventional experimental approach or simulation methods because of its ability to analyze extensive and complex data patterns. ML can also be used to reveal insights into the underlying separation mechanism and find key features that may guide future membrane design for specific applications.^22,23^ The utilization of these algorithms has not only enabled in accurate predictions of material properties, but also expedited the discovery of potential material candidates from a vast search space.^17,24^ Thus, researchers have demonstrated the application of ML to design polymeric membranes for gas separation, nanofiltration (NF), and pervaporation with the shared objective of improving specific performance metrics.^25−27^

The application of AI in material discovery and design of membranes is a relatively new research area, and the number of studies in this domain remains limited to prediction, analysis, and optimization of numerous process conditions. The previously published review papers in this subfield have comprehensively highlighted the advancements in state-of-the-art tools and techniques that assist researchers in applying ML to membrane science and technology.^28−32^ A review positioned at the intersection of the traditional direct design approach and the ML aided inverse design approach is currently absent. We aim to supplement the existing reviews by linking the various substeps involved in building ML models, from a different perspective that is focused specifically on the inverse design of membranes and polymeric material discovery. Researchers have faced several challenges while designing ML models, such as difficulties in formulating strategies to procure, clean, and treat data, as well as identifying key features necessary to support their hypothesis. Additionally, there is a need for adequate representation of categorical data (e.g., polymers, solvents, and ions) in a format that is readable by computers. The black-box nature of ML models requires the use of additional tools to understand the relationship between input features, such as experimental conditions and polymer characteristics, and output features, such as membrane performance. It is of vital importance to introduce comprehensive guidelines on ML methodologies to expedite the discovery of novel membrane materials, particularly from the perspectives of environmental science and engineering.

In this review, we illustrate the advantages and drawbacks of the Edisonian trial-and-error approach and statistical design of experiments (DoE) for synthesis of high-performance membranes. This leads to a discussion on the necessity for data-driven approaches such as ML, where we propose a comprehensive framework, highlighting the various steps involved in the development of ML models. As part of the ML blueprint, we cover the data collection and preprocessing steps, along with various feature generation methods. Subsequently, we briefly discuss various types of ML algorithms used for membrane research, implementation of explainable artificial intelligence (XAI) for model interpretation and validation of the membrane synthesis conditions obtained using ML via experimentation or computational tools. We then review relevant case studies on ML-assisted inverse design material discovery, discussing the research advances made using high-throughput virtual screening (HTVS), Bayesian optimization (BO), and generative ML within a structured framework. Finally, we conclude with a discussion on the constraints and difficulties of current AI applications in this domain, pinpointing existing deficiencies, ongoing progress, future research direction and its environmental implications.

## 2 Traditional Approaches of Polymeric Membrane Material Discovery

The identification of materials and ideal synthesis conditions to fabricate polymeric membranes has been an important area of research for membrane scientists. Polymeric materials are crucial in the advancement of membranes due to their exceptional processability, and widespread availability. These materials will remain vital to membrane technology, as demonstrated by the chemistry-processing-structure-performance paradigm.^33^ The pursuit of improved efficiency, performance, and environmental sustainability is the driving force behind the investigation and development of novel polymeric materials for membrane applications. This undertaking necessitates a comprehension of polymer physics and chemistry, as well as a perceptive awareness of the unique requirements of each application.^34^ The utilization of trial-and-error methods and the DoE framework for experimental design and screening has been pivotal in advancing membrane technology. The selection between these approaches often depends on the specific goals, resources, and constraints of the investigation. The fundamental objective of material scientists is to enhance the membrane design efficiency to shorten the research and development cycle, enabling them to keep pace with rapid advancements in science and technology.

### 2.1 Trial-and-Error Methods

**Figure 1:**
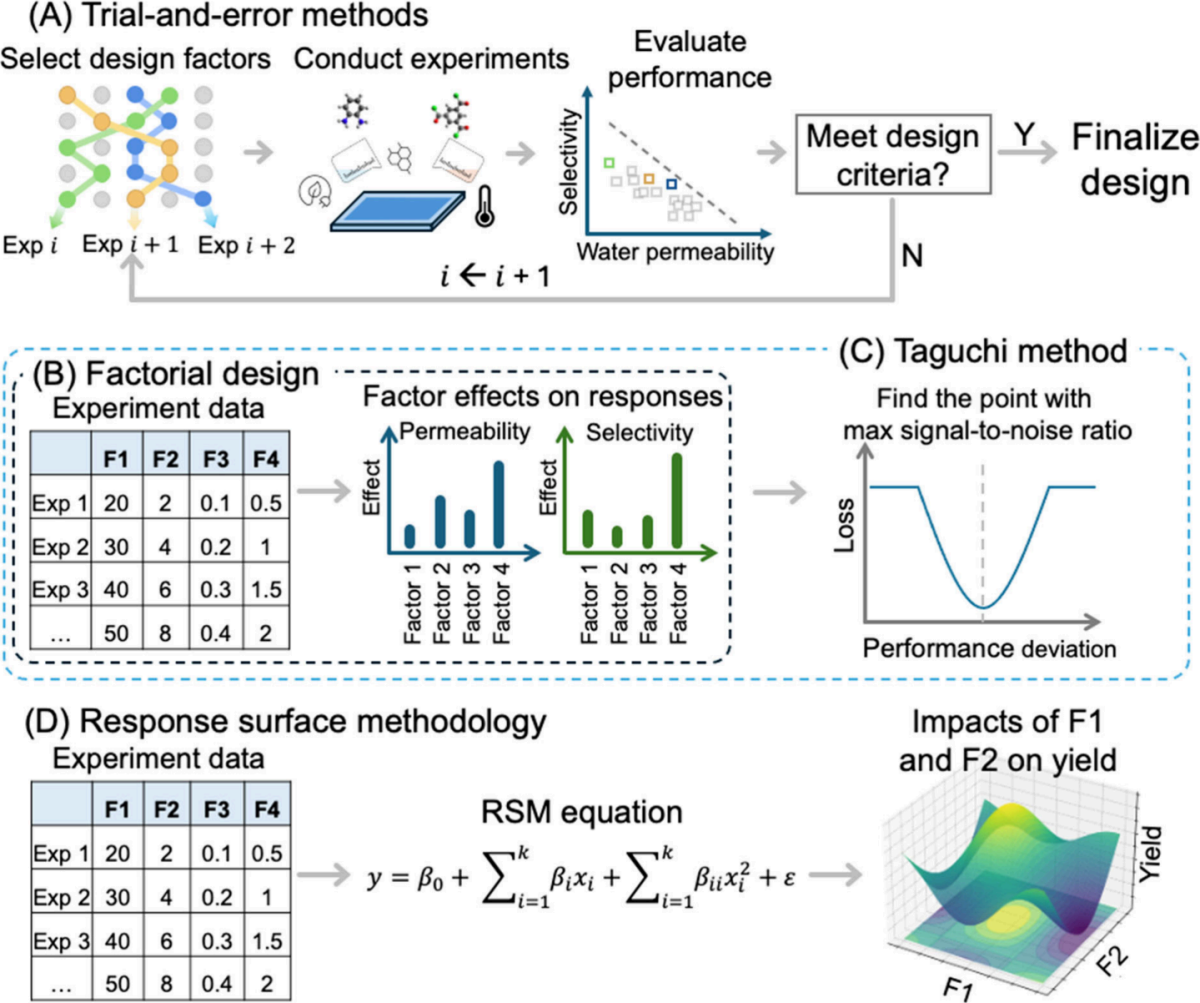
Design and discovery of polymeric membranes by using (A) trial-and-error method and systematic methods including (B) factorial design, (C) Taguchi method, and (D) response surface methodology.

The trial-and-error approach has been the fundamental basis for the evolution of polymeric membrane technology. Researchers using this methodology combine or choose different polymers based on established knowledge or assumptions regarding certain polymer characteristics.^35^ Subsequently, these polymeric membranes undergo a sequence of experiments to assess their appropriateness as potential candidates, with a specific emphasis on performance metrics, such as permeability, selectivity, and stability.^36^ This practical experimentation-based process is extremely iterative, often requiring numerous cycles of synthesis and testing to discover a polymer with desired properties (Figure 1A).

The effectiveness of trial-and-error approaches is greatly dependent on the researcher’s discernment and expertise. Researchers select materials and process conditions that show potential in accordance with their knowledge of polymer chemistry and the desired characteristics of the membrane.^37^ While the trial-and-error approaches are characterized by their simplicity and directness, they are also recognized for being time-consuming, labor intensive, resulting in the squandering of resources. Furthermore, the task of predicting results of trials becomes challenging with a limited comprehension of the correlation between a material’s structure and its performance. The selection of the appropriate approach is contingent upon the particular application, available resources, and desired level of efficiency. While this approach may require significant time and resources, it has the potential to produce unforeseen results, often resulting in significant advancements that might not be attainable through more systematic methods.

### 2.2 Experimental Design and Screening

Although trial-and-error method is still useful due to its simplicity, a systematic approach to design high performance membranes is necessary.^38,39^ The utilization of experimental design methods provides an efficient strategy to synthesize membranes by leveraging statistical tools to optimize experiments, analyze data, and evaluate outcomes.^40^ This enables in a focused exploration of synthesis conditions, pinpointing the key parameters that impact performance and their ideal values in membrane applications.^41^ Although it demands sophisticated statistical expertise and possibly a larger initial investment, this method provides a more profound understanding of the relationships between structure and properties. Consequently, it guides the design of polymeric membranes in a more efficient manner.

The DoE is a comprehensive framework with multiple experimental designs. It provides variety and robustness in organizing, analyzing, and interpreting controlled tests to evaluate the factors that influence the value of a parameter or collection of parameters. It can be tailored to accommodate a broad spectrum of factors and enables a thorough examination of cause-and-effect relationships. Utilizing statistical methods and factorial designs, DoE can effectively minimize the number of required experiments, resulting in expedited and economically efficient research. The field of DoE encompasses a range of methodologies, including factorial designs, Taguchi methods, and response surface methodology (RSM).^42−44^

Factorial design is a fundamental technique in DoE that effectively investigates the impact of individual factors and their interactions, which is essential for comprehending intricate systems (Figure 1B). This approach is especially beneficial when examining a significant number of factors as it offers a thorough understanding of how these variables affect the desired outcome. Scientists frequently utilize factorial design approaches to methodically investigate the impacts of different polymers and additives on membrane characteristics.^45,46^ Identifying viable material combinations and creating a baseline for further optimization is a critical step in this process. Scientists can determine the performance characteristics of membranes by changing elements, such as polymer type, pore-forming agents, and cross-linkers.^39,47^ The Taguchi Method, well-known for its emphasis on robust and resilient design, aims to minimize variability and improve product quality by reducing susceptibility to external noise factors (Figure 1C). The utilization of orthogonal arrays optimizes the experimental procedure, reducing resource requirements while providing significant insights on the impacts of various parameters.^48^ The RSM is utilized for comprehensive process optimization (Figure 1D). This method is highly effective when the relationship between the input factors and the output responses is not well understood. It involves a series of planned experiments to establish a mathematical model that accurately represents a response surface map. This assists in the investigation of the most favorable conditions to achieve the desired outcome.^49^ These methods (i.e., Factorial design, Taguchi, and RSM) are highly valuable in optimizing the synthesis and processing conditions for polymeric membranes. By enabling researchers to pinpoint the most relevant elements and their interconnections, these methodologies enhance the efficiency of membrane development.^50^Table S1 (Supporting Information) provides a brief overview of various traditional methods used for exploring optimal process parameters for membrane design.

### 2.3 Challenges and Limitations of Traditional Methods

Traditional methods for discovering and designing polymeric membranes have largely depended on empirical approaches, particularly the trial-and-error testing of various material combinations and processing conditions. Researchers typically synthesize polymers and then evaluate their membrane-forming capabilities, focusing on properties like permeability, selectivity, mechanical strength, and chemical stability. This often leads to a repetitive cycle, where the synthesis stage is revisited to adjust polymer compositions or processing parameters, continuing until a material meets the desired criteria. On the other hand, DoE can pose challenges due to its complexity, requiring a high level of statistical competence for successful execution. Based on various experimental design methodologies (e.g., full factorial, central composite design, and Box-Behnken), researchers develop an experimental design matrix by fixing one variable and systematically varying the levels of other variables to generate the required experimental combinations. Conducting and evaluating experiments can be labor-intensive, and the cost associated with extensive experimental trials can be considerable, especially in intricate multifactorial designs.^50^ In certain cases, researchers may integrate multiple design approaches to find the optimum response which can lead to increased operational difficulty.^51^ A major drawback of these conventional methods is their unpredictability, making it difficult to predict which polymers will perform effectively as membranes. Moreover, both approaches can be environmentally and economically burdensome, particularly due to the extensive use of chemicals and solvents in polymer synthesis and processing, which could be a constraint for smaller research teams or institutions with limited budgets.^52,53^

The trial-and-error approach often relies on using theoretical equations to model transport and describe the underlying separation principles of membrane processes (e.g., Solution-diffusion, extended Nernst-Plank, Donnan-steric pore model with dielectric exclusion).^54−57^ DoE, on the other hand, develops first- or second-order models to approximate the membrane performance based on the synthesis conditions or membrane properties.^58,59^ These models are easy to interpret, but they struggle to capture the complex nonlinear relationships between synthesis conditions, membrane properties, and membrane performance. To address the limitations of traditional methods, ML algorithms are increasingly integrated into the discovery process of polymeric membrane materials, offering a potential to accelerate innovation and drive a paradigm shift in the field.^30,32,60,61^

## 3 Machine Learning for Polymeric Membrane Material Discovery

**Figure 2:**
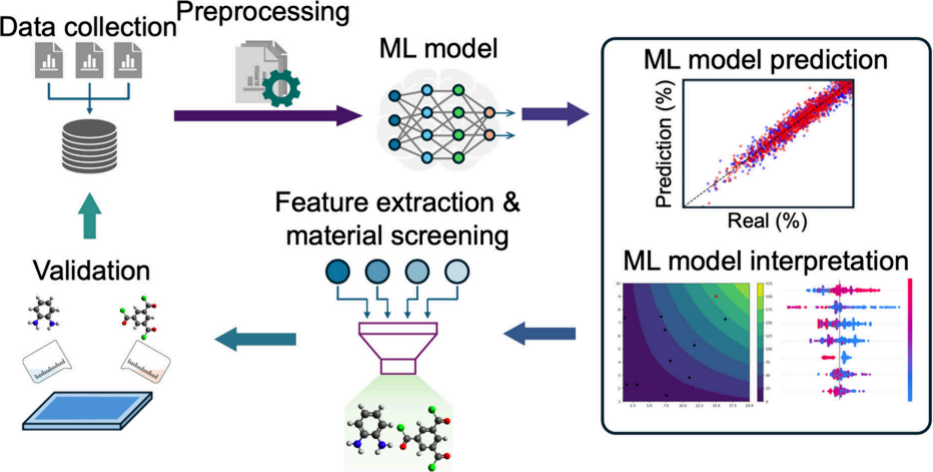
Machine learning pipeline for the inverse design and discovery of polymeric membranes.

Leveraging massive data, ML uncovers the intricate relationships between input variables and outputs, enabling predictions without possessing an extensive domain knowledge.^62^ This approach can surpass the traditional, time-consuming, and costly methods for membrane design, offering a more efficient and cost-effective pathway to innovation in membrane technology. It is impossible to screen all possible polymeric materials for optimizing membranes by using traditional methods given the huge number of polymer candidates, each possessing unique physical and chemical properties. ML has been successfully used to tackle this problem since it enables in rapid screening of materials for membranes, reducing the time and effort required for experimentation or computational assessment.^63^ A typical workflow for the development of polymers using ML approaches is as follows: (1) Data collection: Acquisition of comprehensive, high-quality data is indispensable for building robust ML models that predict membrane performance. Data set for ML can be experimentally generated, extracted from existing literature, or obtained from simulations. (2) Data preprocessing and feature generation: Preprocessing the data collected to handle missing values and outliers. The data can be normalized or scaled to ensure uniformity within the ranges of input variables. Additionally, the structural information from categorical variables needs to be converted into machine-readable representations for ML model use. (3) ML model development: After the data is preprocessed, several ML algorithms can be used depending on the nature of the problem and data characteristics. (4) ML model interpretation: Interpreting ML models helps identify the features that are most important to the model’s performance. This can narrow down the search space for new polymer candidates or find optimal experimental parameters. (5) Validation: Based on the fabrication conditions or polymeric candidates screened using the ML models, we can validate the model either experimentally or through computational tools (Figure 2).

### 3.1 Data Collection

In the context of employing ML for polymer membrane research, the significance of data cannot be overstated. The effectiveness of ML models is significantly impacted by the quality and quantity of data they are trained on. One of the more pronounced challenges in developing robust ML models is associated with the limitation in the available data, especially in maintaining high quality. Smaller, low-quality data sets fail to represent the full diversity (either in terms of the number of data points or the required number of features) of the entire data population, which can result in biased predictions. Training with small data sets often results in overfitting as the model learns noise in the data instead of intended underlying patterns, compromising its ability to generalize to new, unseen data. Larger, high-quality data sets can help overcome these shortcomings by reducing bias, improving feature representation and allowing more room for models to generalize beyond the training data. There is a lack of well-defined criteria to identify low quality data, which makes it difficult to evaluate data reliability. Cross-checking the data can improve the quality of the data set by identifying outliers or unreliable data points. A commonly accepted guideline to prevent overfitting and enhance the model reliability is to ensure that the data set includes at least ten times more data points than the number of input variable types.^64^ Common data collection methods are described in the following subsections.

#### 3.1.1 Traditional Method and Medium/High-Throughput Experimentation

Manual experimentation is a common method to generate high quality data. Experimentation within a closed laboratory environment allows for quality control and standardization, which is critical to the data collection process. The biggest issue with this method is that it is extremely time-consuming and requires a lot of manpower. It is exceptionally difficult to conduct hundreds of experiments and generate a data set. Thus, using this experimental approach to develop a comprehensive data set could take months or years. Glass et al. devised a high-quality data set with only 50 data points to predict water permeance and zeta potential of polymeric membranes. They obtained high coefficients of determination (>0.9) using gradient boosting methods, showcasing the potential of in-house derived data sets in ML.^65^

Medium/high-throughput experimentation has emerged as an effective approach to conduct a vast array of experiments simultaneously or through automated methods. These methodologies are often coupled with combinatorial approaches, where various factors or variables are systematically combined in different permutations to identify optimal operational or synthesis conditions. For example, Cano-Odena et al. used a high-throughput setup (HTS) to find the optimum parameters for the removal of ibuprofen using cellulose acetate-based NF/RO membranes.^66^ The authors were able to complete the entire experimental work within 3 months, including the time required for optimization using genetic algorithms. In another study, Ignacz et al. designed a medium-throughput setup (MTS) crossflow system that enabled them to collect around 1,938 rejection data points measured for 3 membranes and 10 green solvents. This comprehensive data set allowed for an in-depth analysis of membrane performance and selectivity, as well as an exploration of the correlation between the molecular weights and selectivity.^67^ As demonstrated in the aforementioned studies, HTS/MTS has shown its potential in generating extensive data sets for ML, data analysis, and optimization processes.

#### 3.1.2 Data Collection from Literature

The most widely used approach to curate a data set for ML is data mining from literature.^68,69^ Using journal databases like Google Scholar, Scopus, and Web of Science, researchers can manually find publications and articles related to their problem statement for data extraction. This method is much more resource efficient as compared to performing new experiments since it leverages the studies conducted over the past few decades. However, the data found in research papers is often sparse and presented in various formats (e.g., tables, graphs, figures, or text), with no standardized reporting criteria available.

Researchers have used a combination of input variables representing the membrane and polymer properties to predict membrane performance metrics such as water/solvent/gas permeability, salt rejection, gaseous selectivity, membrane fouling characteristics, conductivity, ion selectivity, and ion transport rate for their desired application.^13,26,70,71^ In Table S2 (Supporting Information), we have listed several examples of the different labels used to collect data. Extraction of data requires domain knowledge to navigate through the literature and find the relevant data points. This process is quite tedious and time-consuming since finding relevant articles with all the key features is required to build accurate ML models. The challenges associated with manual data collection have prompted researchers to seek computational (DFT and MD) tools for data generation and natural language processing (NLP) based methods to expedite the data collection process which will be explained in Sections 5.2 and 5.3.

### 3.2 Data Preprocessing and Feature Generation

After data collection, the raw data need to be cleaned and prepared to make it suitable for further analysis or employment in ML models. Data preprocessing or pretreatment has several aspects associated with it. Data cleaning involves the identification and rectification of errors, removal of duplicate entries and outliers from a data set. This is to improve the quality and integrity of data while avoiding any biases.^64^ Data imputation is the process of filling in missing values in a data set with substituted values. This is typically done when a data set has missing values for some of the features, which is usually the case when data is mined from literature. Some researchers use either mean or median values to impute data sets, whereas others utilize data collected in its raw format (true missing values) since these features have physical and chemical properties.^72^ Data normalization is the process of adjusting data distribution to make it suitable for ML analysis. This involves standardizing the data to have a mean of zero and a standard deviation of one and transforming the data to follow a normal distribution. For categorical features, it is important to convert them into computer readable forms as ML models typically cannot process categorical features (e.g., types of polymers, solutes, or solvents) directly.^73^ Examples of these computer-readable forms include one-hot encoding (transforming categorical data into binary vectors), label encoding (assigning each category a unique integer), and binary encoding (a hybrid method that combines aspects of both to efficiently handle numerous categories with minimal data expansion).^16,74^

Feature generation, also known as feature engineering, is a strategic process of creating new features from both numerical and categorical features to improve ML model performance. The goal of this process is to transform (e.g., combination or decomposition) the existing features into more informative and insightful forms.^75^ By using domain knowledge, we can incorporate attributes potentially relevant for predictive analysis, improving the capability of the ML models to discern complex patterns and relationships. For example, water composition such as types of ions in feedwater can influence charge effects, osmotic pressure, and scaling in membrane experiments. Instead of listing all cations and anions with their concentrations, utilizing ionic strength can be more informative and simpler feature for ML. Descriptors are fundamental representations of chemical structures, playing a crucial role in feature generation. Traditional descriptors encompass compositional, structural, and spectral information. In the context of polymer materials, the macromolecular chains consist of repeating units linked by covalent bonds. The properties of materials are directly influenced by the structures and compositions of these repeating units. As a result, the focus of research on polymer descriptors revolves around the characterization of these repeating units.^76^ The following subsections discuss the most frequently used descriptors to represent categorical features.

#### 3.2.1 Line Notation

Simplified molecular-input line-entry system (SMILES) is a widely used format to represent the structure of molecules.^77,78^ SMILES strings consist of alphanumeric characters that represent atoms and bonds in a molecule.^79^ It encodes all the atoms, bonds, rings, and branches of the molecule. An important aspect of SMILES strings is that they can be directly used as a descriptor in ML models or they can be transformed to other descriptors.^80^ Thus, SMILES strings play a crucial role in representing molecules in a format that can be easily processed by ML algorithms, facilitating the application of ML in polymer design and development.

#### 3.2.2 Polymer Fingerprinting

Polymer fingerprints are compact and lossless representations of polymer structures that capture important structural features, such as monomer sequences, branching patterns, and functional groups. Morgan fingerprints (MF) are the most widely used fingerprinting method in membrane design. MF captures the substructure around non-hydrogen atoms within a defined radius and converts the molecules into binary vectors which are suitable for ML model training. The formation of these binary vectors, however, can result in “bit collision” wherein the representation has a single encoding for the multiple functional groups. This means that random substructures without any contribution to the performance get included in the model training process and its subsequent interpretation. Increasing the number of bits (fingerprint size) is one way to tackle the issue, however, it requires higher computational power to process due to increased number of features. A newer method such as molecular access system (MACCS) key vectorization helps address “bit collision” by mapping the specific substructures to individual indices.^81^ Additionally, binary morgan fingerprints can only capture presence/absence of a substructure, not how many times they occur. Count-based morgan fingerprints can tackle this problem since they can count the number of times each chemical bond occurs resulting in a more accurate representation of the polymer.^82^ Molecular embedding and molecular graphs are two other commonly used methods for polymer fingerprinting. Molecular embedding generates continuous vector representations of substructures, allowing for the measurement of similarity between different structures, while molecular graph representations view a molecular structure as an undirected graph, making it particularly suitable for applications of deep learning in polymer materials.^83^

Apart from the standard methods used for polymer fingerprinting, group contribution method has been used to determine the structural groups present in the polymer sets. The basic assumption is that the polymeric properties can be represented as a weighted sum of the individual contribution by its constituents. The Bondi method, the Marrero and Gani method, and the Yampolskii’s method are common approaches that have been used to predict gas permeability of polymeric membranes.^84,85^ The newest toolbox in polymer fingerprinting is inspired by the transformer-based architecture for NLP. Kuenneth and Ramprasad present polyBERT, a novel method that outperforms the traditionally used approaches for polymer fingerprinting.^86^ The authors trained a model on a vast data set of polymer SMILES (∼100 million hypothetical strings), enabling it to understand the grammar and syntax of polymer chemical language. This fingerprinting approach has significant potential for polymer property prediction, polymer structure prediction, and the design of new polymers.

#### 3.2.3 Molecular Descriptors

Molecular descriptors encompass a wide range of features derived from the molecular structure that can be used in ML models.^87^ These descriptors are quantitative representations of chemical compounds that capture various aspects of their chemical and physical properties.^88^ Molecular descriptors can be generated using professional descriptor generation software, such as Dragon, or open-source toolkits, such as Mordred or RDKit. Ritt et al. represented anions using molecular descriptors to study the thermodynamic mechanism associated with the design of single-species selective membrane. They were able to showcase the importance of various molecular features (i.e., structure, polarizability, interactions, electrostatics, macroscopic, confined polarizability, confined interactions, and confined electrostatics) that affected the transport of anion of through a cellulose acetate membrane.^89^ In another study, Ignacz et al. obtained the best descriptors that showcase the effect of different functional groups present in solutes on their rejection in organic solvent nanofiltration (OSN).^90^ Researchers have also converted chemical structure of cationic and anionic groups into extensive molecular descriptors to predict ion conductivity of polymer-based ion-exchange membranes.^91,92^

Quantitative structure activity-relationships (QSAR) and quantitative structure property-relationships (QSPR) have been widely utilized to model the physical and chemical properties of molecules on the basis of their chemical structures.^93,94^ These generated properties can be used as molecular descriptors in ML models since they assist in the feature generation of (1) specific molecules we aim to separate or (2) polymers used to design membranes.^65,95,96^ This extensive representation enhances the ML models and can potentially be used to design membranes for tailored applications.

### 3.3 ML Model Development and Approaches

Once the data set is preprocessed and the relevant descriptors are added to transform the data set, it is ready to be used for training ML models depending on the requirements. Supervised and unsupervised learning have been widely used in the research of polymer membrane design. In supervised learning, the algorithm is trained on a labeled data set to map the input feature to outputs (membrane performance). In unsupervised learning, the algorithm is provided with unlabeled data to find patterns or structures within the data without any predefined model output.^97^ This is in contrast with the traditional design methods or supervised learning wherein researchers focus on performance metrics of the membrane to gain insights and develop a hypothesis. Researchers have obtained unforeseen results using unsupervised learning since they can group high-performance materials based on their properties allowing for efficient exploration of new candidates for their desired application.^98,99^ ML algorithms that are commonly used to model polymeric membranes are provided in Table S3 (Supporting Information).

### 3.4 ML Model Interpretation

After ML algorithms are applied to our data set, the best performing model is selected based on the chosen model performance metric (e.g., mean absolute error, mean squared error, root mean squared error). Due to the “black box” nature of ML models, XAI tools have been employed to understand and interpret the model’s predictions. These tools can identify the influence of features on the model output and present the contribution of each descriptor to polymeric membrane performance which can guide membrane design and material discovery. In the subsequent subsections, we discuss the two most commonly used XAI tools for ML model interpretation, namely Shapley additive explanations (SHAP) and partial development plots (PDP)

#### 3.4.1 SHAP

The SHAP method is a commonly used technique that provides insights into the decision-making process of a ML model. It can find the impact of the polymer features on the membrane performance.^100^ The SHAP values for an input feature x gives the prediction *p* as
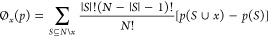
where *S* is the subsets of all features without feature *x*, *N* is the set of all features, *p*(*S* ∪ *x*) are the predictions by the built ML model considering feature *x*, and *p*(*S)* are the predictions without considering feature *x*. The differences among all possible subsets of *S* ⊆ *N\x* are calculated due to the dependency of the effect of withholding a feature on other features in the ML model.^101^

The SHAP values indicate the impact that a specific feature has on membrane performance. Positive and negative SHAP values indicate positive and negative contributions to membrane performance, respectively. Moreover, features with higher absolute SHAP values have greater contributions on the particular performance indicator (i.e., higher influences on the target variable).^102^ Tao et al. correlated the polymer’s functional groups to fractional free volume (FFV) of the membranes using SHAP analysis. They were able to validate the impact of experimentally verified concepts, such as the positive contribution of rigid aromatic rings or phenyl groups to the FFV of polymeric membranes. They also found that carbonyl group density had a positive influence on the transport properties of polymer membranes.^103^ Jeong et al. evaluated whether the knowledge gained by ML for membrane separation aligned with the fundamental principles of membrane science. Using SHAP analysis, the authors were able to reveal the influence of several factors (i.e., the properties of membranes and solutes) on the membrane performance, demonstrating that ML was able to understand the complex mechanisms of membrane separation.^22^ Gallage Dona et al. used SHAP analysis to rank the most important polymer and molecular descriptors for determining the ion-activity coefficients of polymer ion exchange membranes (IEMs).^104^ These coefficients play a crucial role in the ion-transport process across the membrane, directly influencing selectivity in IEMs. In another study, Gao et al. developed an ML model that highlighted the fundamentals of ultrafiltration (UF) membrane design. The authors identified the most significant membrane features and fabrication conditions that need to be optimized for the design of high-performance UF membranes.^72^

#### 3.4.2 Partial Development Plots (PDP)

PDP is a graphical representation showing the relationship between specific features and the predicted outcome of a model while keeping all the other features constant. They help in understanding the marginal effects of a single feature on the output that assists in model interpretation. The average partial dependence function for a feature *S*, *f*_*s*_ can be calculated using
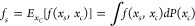
where feature variable *C* is the complement of *S*; *x*_*c*_ and *x*_*s*_ are their feature vectors, respectively.^105^

Wang et al. developed a ML model to screen polymeric materials for pervaporation application. They performed PDP for total flux and separation factor against water contact angle, membrane thickness, Hildebrand solubility parameter, and operational parameters. The PDP studies gave insight on the relationship between the features and their effects on the ML model predictions.^27^ Guan et al. used PDPs to optimize pore size and BET values of MOFs for synthesizing mixed matrix membranes with CO_2_ permeability and CO_2_/CH_4_ selectivity that surpasses 2008 Robeson upper bound.^106^ Deng et al. applied PDP to identify the suitable fabrication candidate space for membranes that exhibited high mono/divalent ion selectivity. They were able to synthesize four new membranes that exceeded the present upper bounds of the permeability-selectivity trade-off.^107^ Researchers also studied the importance of structural and operational features of polyamide nanofiltration (PA-NF) membranes using PDP plots to achieve high water/salt selectivity. The authors determined the ideal ranges of pore radius and zeta potential to achieve high mono/divalent salt selectivity for both anions and cations.^105,108^ Li et al. developed a model to study the permeability-selectivity trade-off on thin film nanocomposite reverse osmosis (RO) membranes. Using bivariate PDPs, the authors identified optimal ranges for nanoparticle loading and nanoparticle size, enhancing both the relative water permeability and relative salt passage for RO membranes. They later used these results as input conditions in their ML model and found improved outcomes in their model-optimized experiments.^109^

Regardless of their results in visualizing the factors associated with exceptional membrane performance, the results from PDP should be carefully evaluated before they are directly applied to experimental design. These plots may produce misleading visuals while extrapolating to regions with little data, often resulting in artificial trends beyond the values at extremities for any specific feature.

### 3.5 Validation

The final step involves validation of the ML models using laboratory experimentation or computational tools. MD is a computational technique that helps in studying the dynamic behavior of molecules and materials at the molecular level. The simulation involves numerically solving the classical equations of motion for a system of interacting particles (atoms or molecules) over a specified time period. It is an important tool in the field of membrane science, facilitating the development of structure–property-performance relationships.^110^

Xu et al. validated an ML model developed to study the permeability and behavior of OSN membranes using both MD simulation and experimental studies. MD simulation was not only able to predict the methanol permeability of polymer intrinsic microporosity (PIM) membranes, but it also provides new insights into their swelling behavior. These membranes were also experimentally fabricated in the lab and tested for their methanol permeability to validate the ML model. The experiments indicated that the PIM-1 membranes had complete solute rejection with methanol permeability close to the predictions of the ML models.^111^ Through their pioneering study, Barnett et al. developed a ML model with a gas permeation data set of 700 polymers. This model was used to predict the gas permeation behavior of over 11,000 polymers and discovered more than 100 polymers exceeding the Robeson upper bound line for O_2_/N_2_ and CO_2_/CH_4_ gas pairs. The researchers validated the results by fabricating two novel polymeric membranes and testing their performance for the separation of CO_2_/CH_4_. The experimental results for the novel polymeric membranes closely matched the predicted values from the ML model, falling within the prediction error margin.^25^ In another investigation, Tayyebi et al. identified the key chemical functional groups which can positively or negatively affect membrane performance using SHAP analysis. They leveraged this knowledge to synthesize grafted PA-RO membranes which was able to surpass the water permeability/salt selectivity trade-off. They further characterized their ML developed membranes using Fourier transform infrared spectroscopy (FTIR), scanning electron microscopy (SEM)-energy dispersive X-ray spectroscopy (EDS), thermogravimetric analysis (TGA) and contact angle measurements which aids in understanding the underlying physical and chemical properties of the ML designed membrane.^112^

## 4 ML-Driven Inverse Design and Material Discovery of Polymeric Membranes

The acceleration of material discovery using data-driven approaches in polymeric membrane research marks a significant shift from traditional trial-and-error methodologies to more efficient design strategies. Using ML as an inverse design methodology will greatly reduce the time and effort required to design and explore new materials to synthesize high-performance membranes.^29,113^ Unlike the traditional trial-and-error method, which is time-consuming and inefficient since it involves testing of candidate materials until those with the desired properties (materials → property) are obtained, inverse design begins by selecting the desired properties of material and then work backward to identify materials that can achieve those properties (property → materials).^114−116^ Inverse-design approach can also be used to simultaneously optimize multiple target properties, a challenge that is often difficult to address using the traditional approaches.^117,118^

The first step of inverse design is to define the scope of the design problem, which involves identifying the input variables (e.g., chemical composition, molecular structure, and membrane fabrication conditions) and specifying the target properties (e.g., permeability, hydrophobicity, and mechanical strength). The next step is data collection and generation, as a comprehensive and high-quality data set is crucial for training accurate ML models. Considering the high computational demand for optimization process, it is important to focus on the most relevant characteristics of polymers and membrane fabrication conditions to define design space, while minimizing the inclusion of less important, irrelevant input variables. To use ML models effectively, the chemical structures of the polymers must be converted into a machine-processable format, as explained in Section 3.2. Recent advancement in polymer informatics plays a substantial role in facilitating these applications by allowing adequate representation of polymers that meet the desired design criteria. ML models which are carefully curated using novel algorithms and feature representations can be interpreted using XAI to provide insights into the underlying principles guiding the separation process. They are also used to identify the desirable physical and chemical properties or membrane fabrication conditions critical to the design of high-performance membranes. Wang et al. developed a ML model to predict the membrane performance of layer-by-layer (LbL) membranes and expedite the exploration of polymer candidates. They conducted SHAP analysis of Morgan Fingerprints which helped in identifying the key atomic groups conducive to high permeability and selectivity. This analysis generated a reference Morgan fingerprint which was mapped against PoLyInfo database using Tanimoto coefficient screen similar candidates.^119^ The authors were able to find 23 potential polymers that can be used to synthesize LbL NF membranes.^120^ In addition to using XAI as a tool for membrane design, scientists employ other methodologies to navigate the vast polymer candidate and fabrication space, such as (1) high-throughput virtual screening, (2) global optimization, and (3) generative ML.

### 4.1 High-Throughput Virtual Screening

Candidates may often be overlooked using the traditional approaches since researchers usually focus on evaluating previously reported high-performance materials or those with similar chemical structures. ML models with high prediction accuracy enable us to curate a high-throughput virtual screening (HTVS) setup that can be used to screen potential polymer candidates.^121^ In a HTVS setup, researchers can define the ideal performance metrics, physical and chemical properties, and functionalities which can be used to screen several polymeric candidates at the same time without conducting any experiments. ML models can predict performance metrics for previously untested candidates, helping narrow down the number of potential candidates from a large selection pool. Yang et al. devised a HTVS setup to identify potential polymeric candidates with a high potential for acetic acid extraction from water using pervaporation. In the first stage, they screened ∼180,000 potential polymeric candidates from the PI1M database (which includes 1 million polymers from Polymer Informatics database) based on their similarity (>0.9) to the polymers used to train their ML model. This was followed up by further screening of the selected polymeric candidates based on predicted permeation separation index (indicating performance) and synthetic accessibility score (indicating ease of synthesis), ultimately identifying 10 potential polymer candidates for pervaporation.^122^ It is also recommended for researchers to not only rely on predefined performance metrics, but also use their intuition, expertise, and knowledge to define better selection criteria and build more robust HTVS setups.^123^

### 4.2 Bayesian Optimization

Global optimization tools such as Bayesian Optimization (BO) have also demonstrated their great potential in a variety of inverse design problems in materials engineering.^124^ BO is an iterative approach that allows the exploration of design conditions using surrogate functions and acquisition functions to build an optimization model to guide membrane design. A surrogate function is essentially a ML model trained on available experimental data, wherein the model can be used to approximate membrane performance metrics based on the input features. This surrogate model estimates membrane performance metrics on the chosen exploratory design space. The acquisition function can then be used to determine which experiments are most likely to be successful.^125^ Gao et al. combined ML and BO to guide experimentation in discovering high performance PA-NF membranes capable of surpassing the current upper bound for permeability-selectivity trade-off.^126^ The surrogate model was trained using the data obtained from synthesis conditions. The BO function was then used to identify new combinations of monomers and fabrication conditions within the input design space. Using these conditions, the authors synthesized 8 membranes that were able to surpass upper bound for the water permeability-salt selectivity trade-off. This validated the ML model’s capability to discover new monomers and synthesis conditions that enhance membrane performance. BO is not only limited to experimental design, but it can also assist in the material discovery process. Chen et al. used a Bayesian algorithm to modify existing polymers within a data set to discover 200 new polymers showing exceptional separation performance for CO_2_/CH_4_ and CO_2_/N_2_.^127^

Given the large number of influencing factors on membrane design, new methods have been developed to apply BO to these high dimensional parameter spaces, while minimizing the computational demands. Gui et al. proposed a taking-another-step BO (TAS-BO), which offers a simple-yet-effective strategy to tackle high dimensional BO problems. At each iteration, a local Gaussian process (GP) is trained using points neighboring the current candidate. This coarse-to-fine local search enables a more efficient exploration of the search space.^128^ A strategic optimization approach can also enhance the efficiency of the discovery process. Dalal et al. used a batch BO to efficiently explore a vast design space of over 5,790 polymer formulations for optimizing pDNA and CRISPR-Cas9 ribonucleoprotein delivery.^129^ The BO predicted the most promising formulations in sequential rounds, significantly reducing the design space. After three rounds of optimization, they sampled less than 10% of the design space while identifying the top-performing polymer combinations for delivery efficiency and cell viability.

### 4.3 Generative ML

Generative ML techniques, such as RNNs or graph-based design tools, can also be used to navigate the chemical space and accelerate material discovery by generating new data points based on previously trained data.^130−132^ In general, high dimensional polymeric data is scaled down to a lower dimension to capture relevant features, which are then used to generate new polymeric candidates.^133^ These candidates can be tested using accurate ML models built on training data, enabling us to screen newer high-performance materials. Yang et al. trained a ML model with 778 polymers mapping their Morgan fingerprints to their gas permeabilities.^134^ This helped them develop an accurate ML model for predicting the permeabilities of 9 million new polymers that had never been tested before for gas separation. These 9 million polymers were generated using (a) RNNs trained on SMILES strings of existing polymers, (b) theoretical chemical reaction between binary pairs of dianhydride and diamine which yields polyimides, and (c) ladder polymers generated using a combination of monomer combinations and RNN generation. They used the ML model to predict the permeabilities of these polymeric membranes to identify new candidates surpassing the Robeson upper bound. In another study, Giro et al. developed an inverse-material design workflow to design new monomers for carbon capture with targeted property ranges for the permeability of CO_2_, glass transition temperature, and half-decomposition temperature. They represented the input molecules as feature vectors (containing encoded information related to molecular building blocks of the monomer) whose features were extracted using regression modeling. These feature vectors were optimized using Particle Swarm Optimization and converted to molecular structures using a graph-based McKay’s Canonical Construction Path Algorithm to generate new polymeric structures with desired properties.^135,136^ Even though genetic algorithms (GA) are not traditionally classified under the generative ML umbrella, researchers generated new polymer compounds using them based on the chemical fragments present in the polymer data set.^137^

Despite the unforeseen performance showcased by the hypothetical candidates engineered using these generative techniques, their synthesis can be quite complex which limits its application. Including synthetic accessibility score within the screening or design process is one way to tackle this problem, however, more research regarding their synthesizability is desired.^123^ Another major factor that governs the use of generative ML models is the requirement for high-quality data sets to capture the relevant features. Poor quality in training data would result in the formation of unrealistic samples.^28^

In Table 1, we explore a series of success case studies using ML to enable the discovery and design of new polymeric membranes, organized within a comprehensive framework. These case studies aim to provide an overview of the synergy between ML and polymeric membrane technology, highlighting current advancements and its future potential in our field.

**Table 1:** Case Studies Demonstrating the Application of ML for Polymeric Membrane Material Discovery

data collection	feature generation	ML algorithms	model interpretation	validation	inference	ref
Around 500 to 1000 data points for 6 gases (CH_4_, CO_,2_, He, H_2_, N_2_, O_2_) from the literature	Polymers represented as binary fingerprints	Gaussian process regression (GPR)	-	Synthesis of 2 polymers with performance surpassing the current upper bound for CO_2_/CH_4_ separations.	Predicted the gas permeabilities of ∼11,000 potential polymers and screened candidate polymers whose predicted permeabilities lie above the Robeson upper bound.	(25)
Polymer chemistry and gas permeability data from PoLyInfo and Membrane Society of Australia (MSA)	Polymers represented as SMILES strings. Their molecular representations were performed using Morgan Fingerprinting and Chemical Descriptors.	Random Forest (RF) and Deep Neural Networks (DNNs)	SHAP analysis revealed the chemical basis required for overcoming permeability selectivity trade-off.	MD simulations were used to verify the permeabilities used to predict the ML model.	ML models were used to conduct high-throughput screening of over 9 million hypothetical polymers with unknown gas permeabilities designed using generative ML.	(134)
152 different data points collected from the literature for OSN	Molecular descriptors (representing chemical structure of polymer and solvent) or gross descriptors (representing important measurable polymer and solvent properties)	Kernel Ridge Regression (KRR), Gradient Boosting Regression (GBR) and LASSO	SHAP analysis found that the presence of hydrophobic and amine fragments improved solvent permeability.	MD simulations were used to provide insights on swelling behavior while PIM membranes were synthesized for experimental validation.	Study showed the applicability of ML, combined with molecular simulation and experimentation, to predict solvent permeability and develop new polymeric membranes.	(111)
Data collected from 218 publications for PA-NF membranes	SMILES and Morgan Fingerprints to represent polymers. The salts were represented using molecular descriptors.	XGBoost and CatBoost	The authors developed a reference Morgan Fingerprint using positive contributions of the atomic structures obtained using SHAP to screen 20 new monomers.	The models were validated by synthesizing 8 PA-based NF membranes.	Showed the applicability of Bayesian optimization to design new experiments and explore the fabrication search space to design high-performance NF membrane materials.	(126)
Generated data (114 data points) from their own experiments	The synthesis conditions for PA-TFC membranes as input features	ANN	PDP plots defined the fabrication candidate space for desirable performance.	Synthesized 4 PA membranes according to the ML model showing high mono/divalent ion selectivity.	Devised a data driven methodology to synthesize high-performance PA-TFC membranes that exceeded the upper bound of the permeability selectivity trade-off.	(107)
Gas permeability data set consisting of 1,169 homopolymers collected from patents and publications	Polymers represented as SMILES. Topological, Geometrical and Structural descriptors were used for property predictions.	LASSO regression, Ridge regression, Elastic Net Regression, RF, KRR, SVR	-	MD simulations were used to model CO_2_ permeability for ML model verification. It also gave insight on the filtration dynamics of materials.	Suggested a framework for ML-based generative monomer design for carbon capture.	(135)
The data was collected from 30 publications with 227 different types of LbL membranes	Numerical features, such as concentration of polyanions and polycations, reaction time, ionic strength, along with categorical features including LbL method type, polyanion and polycation types, and the name of substrate, serve as the input features.	RF, Boosted tree model, Linear Regression, and XGBoost	SHAP analysis of Morgan Fingerprints gave insights into the key chemical structures important for membrane performance.	-	Designed 2 reference Morgan Fingerprints on the basis of the contributions obtained from SHAP to give 23 potential polymers candidates for LbL application	(120)
1,347 experimental data points collected from 41 journal articles.	The input features include SMILES along with 5 molecular fingerprinting techniques.	GBR	PDP and SHAP analysis revealed that operating conditions are the most important for permeance whereas molecular weight of solutes are the most important for rejection.	Synthesis of a TFC membrane with good agreement between actual and predicted permeance for 3 solvents (dimethylformamide, methanol, and acetone)	In-silico design of potential membranes synthesized by combining different monomers was conducted. The performance of these membranes was predicted to screen potential candidates for OSN application.	(138)
Data collected from^126^	SMILES notation and Morgan Fingerprinting	CatBoost and RF	SHAP analysis used to identify chemical functional groups important for membrane performance to select additives for membrane grafting.	Synthesized and characterized high-performance membranes using FTIR, SEM, TGA and EDS.	This paper showed the application of SHAP to guide the chemical modification process of RO membranes for performance greater than the upper bound for water permeability- salt selectivity.	(112)
∼2400 data points (consisting of 52 unique polymers and 32 types of organic solvents) collected from 264 articles	Morgan Frequency Fingerprint (MFF), which includes topological features in addition to the substructures present in the molecule.	CatBoost, extra trees regression, RF, LGBR	SHAP summary plots were used to reveal the importance of different chemical features which were transformed to principal components.	-	High-throughput screening to identify potential candidates from PI1M data set with the potential for acetic acid extraction from water having high permeation separation index and low synthetic accessibility score	(122)
Collected literature data containing over 780 unique polymers for CO_2_, N_2_ and O_2_	SMILES notation for polymer representation. Extended Connectivity Fingerprint (Morgan Fingerprint) and MACCS for polymer fingerprinting	SVR, K-Nearest Neighbors, Decision Tree and RF	-	Used polymer genome software to make permeability predictions for the hypothetical polymers^139^	Used genetic algorithms to develop new polymers which were screened using a HTVS approach with exceptional permeability and CO_2_/N_2_, CO_2_/O_2_ selectivity.	(137)
Data consisted of 749 Anion Exchange Membranes (AEMs)	SMILES notation was used to represent the copolymers and OH^–^ conductivity, water uptake, swelling ratio (%) and tensile strength chosen as performance metrics. They calculated theconductivity- dimension stability trade-off (CDST) coefficient for AEMs to characterize performance.	XGBoost performed the best out of 12 algorithms used for training	SHAP analysis revealed that octanol–water partition coefficient of cations, number of rotatable bonds in backbones and BalabanJ are the most important parameters for OH^–^ conduction and CDST.	-	The authors screened 2519 potential copolymers from ∼172,000,000 candidates to synthesize high-performance AEMs	(123)

## 5 Recent Progress, Future Directions, and Perspectives

### 5.1 ML Algorithms for Polymeric Membrane Technology

The separation and purification performance of membranes depends on a variety of factors including synthesis conditions, operational conditions, and the structural, chemical, and functional properties of membranes, solvents, liquids and gases. Due to the complexity of this process, researchers are trying to develop new, robust ML algorithms to predict the properties and performance of the polymeric membranes, which can provide a better understanding of their separation mechanisms.

Graph neural networks (GNNs) are a type of neural network designed to operate on graph-structured data, where graph consists of nodes (representing entities) and edges (representing the connections or relationships between these entities). GNNs leverage the inherent structure of graphs to capture the relationships in the data and does not require extensive feature engineering or representation design.^140^ Ignacz et al. used GNNs to model solute–solvent-membrane interactions and understand the structural impact of solutes and solvents on the performance of OSN membranes. With the help of GNNs, they were able to visualize the effects of functional groups and substructures and further extract the atomic and bond level information on the molecules of interest.^141^ Queen et al. also used GNN to develop POLYMERGNN, a model that allows for the prediction of polymeric properties.^142^ In an attempt to improve the standard deep learning model, Li et al. developed a three-component residual ANN (R-TNN) to study the water permeability and salt selectivity trade-off in TFN-RO membranes. Using this approach, the authors were able to adjust the model such that the first two networks emphasized on learning the data from relative water permeability and relative salt rejection, while the other layers focused on feature analysis and gaining knowledge from the previous networks. The authors demonstrated that this modified network outperformed neural networks and ML models (RF, K-nearest neighbor, XGBoost, and adaptive boosting trees) in predicting membrane performance.^109^ Cui et al. combined MD simulations with density peak clustering algorithm based on unsupervised learning to model the ionic channels of membranes. They were able to visualize and quantify the properties of the ionic channels which helped them study water transport across proton exchange membrane fuel cells.^143^ Transformer models have also gained huge attention for the property predictions of polymers. Xu et al. developed TransPolymer, a transformer-based language model to predict the various properties of polymers, which include their electrolyte conductivity, electron affinity, ionization energy, and refractive index.^144^ Zhong et al. used generative pretrained transformer (GPT) based models to develop QSAR relationships for water contaminant properties using SMILES strings. These models outperformed CatBoost-based QSAR models.^145^ The algorithms mentioned above can improve data visualization and membrane performance predictions allowing for better research outcomes.

Integrating ML models with physical and chemical principles can further improve the prediction accuracy of the models and deepen our understanding of membrane separation. Rehman et al. developed a physics-informed deep learning model to study ion transport across polyamide membranes. They integrated charge conservation laws into the deep learning model, which led to an improvement in the prediction of membrane performance.^146^ Lee et al. also developed a physics-informed ML model to study the diffusion of gases through polymeric membranes. Using physical equations, the authors enforced the neural network to learn the physical relationships governing the diffusion process for the prediction of gas diffusivity.^147^ In another study, Wang et al. used physics-informed performance metrics (fractional free volume and average void size) to assess the gas separation of polymeric membranes. They were able to screen polyamide membranes that exceeded the Robeson upper bound plots for several gaseous mixtures.^148^ Researchers have used chemistry informed ML to find promising candidates for solid state polymer electrolytes for lithium-ion batteries.^149^ Thus, incorporating the essential rules of physics and chemistry has the potential to enhance the “intelligence” of ML models and can be of significant importance in utilizing ML to identify new polymers.

There is a computational cost associated with obtaining data from different sources: High-fidelity data have better accuracy and are more expensive to obtain, whereas low-fidelity data are less accurate, but require a lower computational cost.^150^ Multifidelity models use data from multiple sources to address the trade-off between fidelity and computational demands, helping develop accurate ML models with minimal resource use.^151^ Rall et al. developed a multiscale optimization framework that integrates high-fidelity ion transport models with ML to optimize membrane processes for water treatment.^152^ Using the data generated from the one-dimensional extended Nernst–Planck ion transport model, ANN was trained to predict the performance of NF membranes and served as a surrogate model of high-fidelity model. The authors integrated this surrogate ML with mechanistic process models to optimize membrane synthesis properties and overall process design for membrane plant, reducing computational resources and maintaining the accuracy of the physical model. Instead of using ML as a surrogate for high-fidelity models, models across different fidelity levels can also be integrated for inverse membrane design. Lazin et al. suggested an efficient multifidelity BO for solving inverse problems in the quantum control of time-dependent system. By combining low- (prior distribution) and high-fidelity (posterior distribution) models for GP, this method enables efficient exploration of the next query point in BO, reducing computational time and maintaining high accuracy in the optimization process.^153^

Generative ML can innovate and accelerate material discovery by expanding the diversity of potential materials used to design membranes. Generative adversarial networks (GANs) can generate synthetic data with target properties by exploiting sequential or graph representations of organic materials.^130^ Several researchers also used transformer based models to generate unique molecules using SMILES strings and desired property values as inputs.^154−156^ Diffusion models are the latest advancement in generative ML for a variety of chemistry and drug design applications.^157,158^ Inspired by nonequilibrated thermodynamics, these models are able to generate 3D molecular structures through forward and backward diffusion processes. These models captured the chemical and physical properties of molecules represented via graph structures to design target molecules.^159^ Park et al. developed ZeoDiff relying on diffusion model architecture to generate porous materials with user-desired characteristics.^160^

A big challenge in the application of ML on polymeric membrane design is the adequate representation of polymers and additives. Polymer structures are highly complex that have dynamics ranging from various length and time scales.^161^ ML for polymer design requires encoding polymers in formats that are interpretable by computers. The chemical representation technique based on SMILES uses a single molecular representation to extract all the features from the polymer.^162^ The majority of research in this area is still primarily concerned with the topology of individual monomers or cross-linkers, whereas a polymer network might consist of topologies or structural features that are never observed in monomers or cross-linkers. For example, when two different monomers were considered to react to form a new polymer, the newly generated topologies may not be fully defined by the individual monomers alone, and thus the new structure requires further description.^63^ BigSMILES has recently emerged as an extension to SMILES, which is tailored specifically to polymeric systems. It can help in better representation of homo-, co-, and block polymers and map out the nature of the polymer in terms of its branched, network, and terminal group information via bond descriptors, making it an ideal choice for polymer representation.^163^ Researchers have often used nanomaterials such as graphene oxide, titanium dioxide, and carbon nanotubes as additives to modify membranes. These materials are highly complex in terms of their physical and chemical properties, yet researchers usually represent them as categorical features in ML models which may lead to oversimplification of their effects on membrane performance.^26,72,164^ The development of more robust techniques to represent these additives can allow algorithms to learn more nuances associated with their impact, aiding in the development of more accurate ML models.

Another significant challenge in the process of developing ML models is data management, data preprocessing, and data scarcity. Certain models, including DNN, are incapable of processing data sets that have missing values. Filling in missing values with generated data, such as utilizing mean or median values derived from statistical distributions or ML models, can lead to a loss of some of its useful practical insights about the model’s performance.^64^ Yuan et al. imputed gas permeability data in the polymer gas separation membrane database using a multivariate imputation by chained equations (MICE) method, which predicts missing permeability values through an iterative process via predictive models. The imputed data was used to identify promising polymeric materials for applications different from those for that were initially intended.^165^ Data augmentation can expand the size and diversity of training data sets to address data scarcity commonly observed in polymer and membrane science.^166^ Tayyebi et al. generated 300 new SMILES strings by randomizing atom ordering, increasing their data set size from the original 583 points to a total of 17,500 data points.^112^ This data augmentation strategy helped the model train on different representations of the same molecule, enhancing its ability to grasp the chemical space limitations present in the data set.^167^ Transfer learning can also address data scarcity by transferring knowledge from a model pretrained on a larger, related data set for new tasks.^168^ Using transfer learning for a small data set of 12 membrane electrode assemblies (MEA), Tan et al. investigated the influences of anode catalyst ink formation on low-iridium membrane assemblies. By combining transfer learning with the Harris Hawk optimization, the authors significantly reduced experimental cost and achieved high-performance MEA, demonstrating the potential of transfer learning for materials optimization with small data sets.^169−171^

### 5.2 Data Generation Using Computational Tools

A major obstacle in designing polymeric materials using ML for membrane application arises from the diversity of polymer properties, which requires a substantial and high-quality data set for precise modeling. MD simulations and DFT are commonly used techniques implemented to simulate the behavior of polymeric materials at the molecular scale, assisting in the development of membranes with customized properties.^172^ Wei et al. investigated the diffusive response of water in cross-linked polyamide membranes using MD simulation. Their results were consistent with the experimentally obtained flux values, suggesting that MD simulations can reliably surrogate for laboratory-performed experiments.^173^ On the other hand, DFT assists in quantum mechanical calculation of the interactions between polymeric surface and salts or gaseous molecules. This can provide fundamental insights into the separation process and further help in the synthesis of selective membranes.^174,175^

Owing to the improvements in computational power, it is possible to run parallel computational simulations to generate data, which can be used for ML modeling. High-throughput computations have been used to generate vast databases to determine the crystalline and optoelectrical properties of polymers.^176,177^ A scalable modeling and rapid theoretical (SMART) calculation approach has been presented that aims to combine high-throughput calculations with ML for the development of superior carbon capture materials.^178^ This approach can be translated toward the ML process for the development of membrane materials. Tao et al. utilized high-throughput MD to simulate a large data set consisting of over 6,500 homopolymers and 1,400 polyamides to develop a ML model that determines the FFV of polymers.^103^ Researchers used grand canonical Monte Carlo (GCMC) and MD simulations to train ML models with the capability to model the gas separation behavior for binary gas mixtures in mixed matrix membranes.^179,180^ Meng et al. generated a data set containing 2D graphene-based membranes using CALYPSO, a structural prediction tool based on particle swarm optimization. Using this data set, the authors were able to screen membranes for desalination with superior flux, salt rejection, and mechanical properties.^181,182^ Zhang et al. augmented a polyamide NF data set having 10^2^ points to 10^4^ points by using a combination of vibrational augmentation and DFT calculations. They considered the 3D geometry of the monomer structure along with the chemical coupling of functional groups to spatially represent the monomer groups.^183^

Membrane selectivity is impacted by the molecular interactions between polymeric membrane functional groups and targeted species (solutes or gases). DFT models are often used to calculate the binding or adsorption energies which are commonly used to study this interaction behavior. With the advancement of high-throughput DFT, these interactions can be calculated to be used as input features to train ML models. Inclusion of these interactions can potentially aid in the design of selective membranes that allow favorable transport of targeted species. DFT calculations can also assist in modeling energy barriers associated with multiple solute selectivity, further helping in the design of ion selective membranes.^89,184,185^ Thus, data generation using high-throughput computations holds significant potential for enhancing ML process and assist in the discovery of a superior class of polymeric membranes for various engineering applications.

### 5.3 Data Extraction Using NLP and Large Language Models (LLMs)

Scientific research is growing at an unprecedented rate with thousands of publications, reports, and papers being published every year related to polymer, material, and membrane science. It is extremely difficult for scientists to keep up to date with these advances. NLP has emerged as a facilitative approach for information retrieval from literature in the past couple of years.^186,187^ It enables a computer to understand, interpret, and generate human language, bridging the gap between human communication and digital data processing.^188^ NLP facilitates the extraction of data from written texts in diverse formats, enabling efficient analysis and interpretation of large volumes of information. Information retrieval is the first step of NLP, which involves collection of papers of interest as PDF, HTML, or XML files.^189^ Once the papers are obtained, the next step is to process the documents by cleaning up their text to remove irrelevant content and special characters. This step is followed by tokenization wherein the cleaned text is converted into individual names or broad concepts, which are suitable for NLP. Following tokenization, several methods can be used to extract data from the input text. Named entity recognition (NER) technique is utilized to focus on the identification and classification of entities within the text. This process is followed by word embedding wherein the text is transformed into word vectors that can be used for further information extraction.^190^ LLMs such as GPT, BERT, or LLaMA are the latest advancement in this field and have shown great potential for information retrieval. Unlike NER, which is a multistep process requiring intermediate processing and classification of links between entities, LLMs can directly be used to transform input text into structured output data (as JSON documents or other hierarchical structures), allowing for ease in the data mining process.^191^

With the rapid advancement of NLP in the field of material science, there have been multiple instances where it is used for information retrieval.^192−195^ Shetty et al. leveraged this growth in NLP and applied this knowledge to the field of polymer science as they extracted and processed data from ∼0.5 million publications.^196^ They trained word vector models on the polymer literature corpus, encoding polymer domain knowledge in the vector space. The data extracted from the literature can facilitate the generation of training data for downstream ML models. In this study, unsupervised ML was used to identify application trends and generate meaningful information. The authors were able to establish relationships between monomer-polymer as well as property-polymer which helped them cluster polymers based on their properties (e.g., conductivity, biodegradability, and adhesiveness). Additionally, the authors demonstrated the capability of the model to predict polymers with new functions. For example, a model trained on a subset of data for a particular year could accurately predict the occurrence of the polymer for a completely different application in the subsequent years. This highlights the potential of NLP in polymer science, allowing researchers to uncover new insights and applications from a vast corpus of literature. Thus, by leveraging the data extracted and processed through NLP techniques, researchers can build more sophisticated ML models, enhancing the ability to derive meaningful insights and prediction from large data sets in polymer science.

Developing a fully automated, end-to-end ML model that regularly updates its data set from recently published literature through NLP will revolutionize the field. However, this comes with its own set of challenges, the biggest of which is to develop a pipeline that can effectively embed textual, written, or graphical data points into the correct features, especially in cases with large dimensionality.

### 5.4 Collaborative Efforts and Open Data Sharing Initiatives

Given that data serves as the fundamental basis for ML, it is crucial to address data-related challenges, such as the scarcity of adequate high-quality data or metadata. Various techniques can be employed to acquire data, including manual collection, high-throughput experiments or simulations, NLP, curated databases, and user populated databases. Each of these methods has its own specific factors to be considered.^197^ Manual data collection is tedious in nature, while high-throughput experiments/simulations require specific expertise and can be time-consuming, resource-intensive, and interdependent.^198^ Open access databases for polymeric membranes can offer a solution to the issues of data acquisition. These platforms play a crucial role in enabling the prediction and analysis of polymer properties, addressing the data scarcity problem by organizing extensive data sets from various sources.

**Figure 3:**
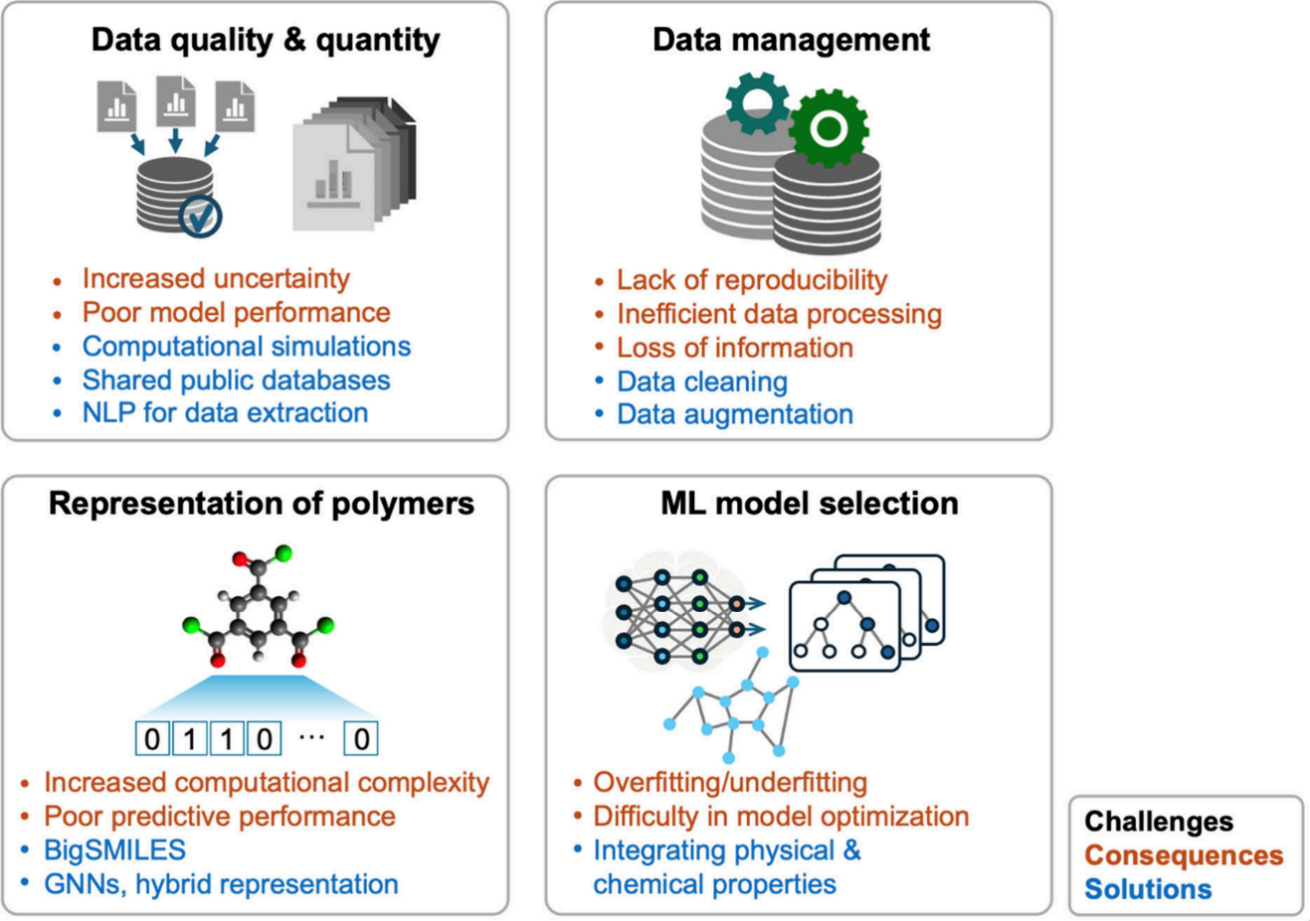
Challenges, consequences, and solutions related to the application of AI for polymeric membrane discovery.

The Open Membrane Database (OMD) is a great step for a collaboratory database in the field of membrane science, which allows researchers to create a centralized archive for thin-film composite RO membranes for water purification and desalination purposes. It has data from over 1,000 different types of polymeric membranes from peer reviewed journals and patents.^199^ MSA and PoLyInfo consist of gas permeability data for at least one of the gases among He, H_2_, O_2_, N_2_, CO_2_, and CH_4_ for around 800 homopolymers. These databases have already been used in ML studies to model the gas separation of polymeric membranes.^134,165^ Additionally, OSN database is a repository which contains a collection of publications with their data sets for membrane applications such as NF, RO, and gas separation.^200^ In the future, it is encouraged that researchers upload their data sets along with their publications as supplemental files or web sites (e.g., GitHub, Zenodo, and Figshare) facilitating convenient access and retrieval of experimental data.^30^ By sharing data, resources, and expertise, authors can develop comprehensive databases containing information on material properties, synthesis methods, and performance metrics. Open data sharing initiatives foster innovation, reproducibility, and transparency by granting access to high-quality data sets for training and validating AI/ML models to researchers worldwide. Figure 3 gives a summary of the commonly faced challenges during the application of ML tools for membrane design, how they impact model development process, and potential approaches of dealing with these problems.

## 6 Environmental Implications

The current research paradigm for membrane material discovery and development is largely driven by the direct design approach. Experimentally testing new polymeric materials is costly, resource-consuming, and challenging, which significantly slows down novel membrane design process. ML-aided design strategy largely relies on capturing key chemical structures with positive contributions to performance, which are used to screen top polymer candidates. This general-purpose framework can be applied to discover materials for environmental applications such as gas separation, water purification, energy generation, solvent and other resource recovery, carbon capture, which has significant real-world impact.

One of the biggest open challenges in the field is the development of polymeric membranes with high selectivity. ML aided-inverse design approach has provided a fit-for-purpose strategy to synthesize polymeric membranes with exceptional selectivity. The ability to tailor membrane selectivity depending on the application can assist in the removal of contaminants and micropollutants from wastewater, or the recovery of critical industry essential resources such as expensive solvents, nutrients, and minerals.^201,202^ Using high-performance materials for pollutant removal from wastewater can improve process efficiency, reducing the reliance on chemical treatments and minimizing the release of contaminants into aquatic ecosystems.^203,204^ With the advancement of computational data generation tools, researchers have the potential to tackle pressing issues such as the recovery of plant essential nutrients or critical metals with minimal reliance on experimental data. AI-driven membrane design enhances the efficiency of carbon capture and industrial gas purification, contributing to lower greenhouse gas emissions and improving sustainability efforts across energy-intensive industries.^205^ Leveraging ML to screen candidates and design membranes can minimize material waste and reduce energy consumption through process optimization, which reduces the environmental footprint of membrane design and operation. Developing robust quantitative metrics to relate the chemical structure of polymers to its biodegradability can aid in greener synthesis routes; however, research in this area is still in its infancy.^206,207^ Researchers need to ensure that the environmental footprint of membrane processes is minimized throughout their lifecycle, from production to end-of-life disposal. These advancements align with the broader goals of the circular economy by optimizing resource recovery and minimizing waste.^208^

It is not implied through this review that the direct-design approach is inferior to the ML-aided inverse-design approach. ML research benefits from the data generated through extensive experimentation as much as traditional experimentalists can benefit from a guided approach. The computational resources required for ML model development and high-throughput data generation are energy-intensive, potentially offsetting the environmental gains realized through optimized membrane performance and design. Broader adoption of ML in membrane design may require comprehensive life cycle assessments to ensure that the energy and material savings during membrane operation outweigh the carbon footprint incurred through AI computation.^209^ Interdisciplinary cooperation among material scientists, chemists, physicists, computer scientists, and environmental engineers is crucial for addressing the issues and promoting innovation in the design and discovery of polymeric membranes in an environmentally sustainable way.

## Supporting Information Available

Details on traditional methods used for polymer design for membranes, examples of input and output features used for ML in polymeric membrane research, and commonly used ML algorithms in polymeric membrane research (PDF)
