# Supplementary material for: Machine Learning-Aided Inverse Design and Discovery of Novel Polymeric Materials for Membrane Separation
Source: Environ Sci Technol. 2024 Dec 16;59(2):993–1012. doi: 10.1021/acs.est.4c08298 (PMC11755723; doi:10.1021/acs.est.4c08298)
Supplement: Supplementary file 1 — es4c08298_si_001.pdf [file es4c08298_si_001.pdf]

Machine learning-aided inverse design and discovery of novel polymeric materials for membrane separation

Supporting Information

Environmental Science & Technology

Raghav Dangayach [a,#], Nohyeong Jeong [a,#], Elif Demirel [a], Nigmet Uzal [a,b], Victor Fung [c], and Yongsheng Chen [a,*]

[a] School of Civil & Environmental Engineering, Georgia Institute of Technology, Atlanta, GA, 30332, United States

[b] Department of Civil Engineering, Abdullah Gul University, 38039, Kayseri, Turkey

[c] School of Computational Science and Engineering, Georgia Institute of Technology, Atlanta, GA, 30332, United States

[#] Authors contributed equally to this work

*Corresponding authors:

Yongsheng Chen: yongsheng.chen@ce.gatech.edu

Number of Pages: 7

Number of Figures: 0

Number of Tables: 3

Table S1. Traditional methods used for polymer design for membrane applications.

Membrane	Application	Methodology	Implications	Ref
Polyaryl nanofilms on PES supports	NF	Trial-and-error	Developed a highly permeable membrane without compromising on the solute selectivity. Authors used their domain knowledge to incorporate polymers of intrinsic microporosity (PIM) in the thin separating layer to create free volume.	1
Vinyl-addition-polymerization polynorbornenes with imide groups	Gas separation	Trial-and-error	Developed highly permeable CO_2 membranes with good selectivity by introducing fluoroaryl groups in the membrane structure that broke the Robeson upper bound for CO_2/CH_4 mixed-gas separation.	2
PVA/PAN membranes	Pervaporation	Trial-and-error	The effects of concentration of PVA, glutaraldehyde (GA), hydrochloric acid, filtration time and transmembrane pressure were studied for ethanol dehydration.	3
Attenuated thermal-regulated phase inversion membranes	NF	Trial-and-error	Alteration of temperature during the membrane synthesis process was studied to show how it influences the membrane performance.	4
PVC/Pebax TFC membranes	Gas separation	Factorial design	The objective of the study was to determine the most effective synthesis conditions maximizing the CO_2 permeability and achieving excellent selectivity.	5
PSF	Ultrafiltration (UF)	Factorial design	Used a 2^k factorial design to synthesize membranes showing exceptional water permeability based on polymer-solvent-time relationships.	6
Hollow Fiber CA	RO	Taguchi method	Several process factors were considered to predict the optimum conditions for the design of hollow fiber CA membranes. Experimental verification results revealed similar results between the predicted performance and the actual performance.	7
PES membranes	UF	Taguchi method	Membrane synthesis parameters such as polymer concentration, additive concentration, additive molecular weight, and coagulation bath temperature were optimized to find the best conditions for exceptional membrane performance.	8
PES/PVP	Ion Exchange Membrane (IEM)	Taguchi method	Taguchi method was used to study the effects of the ratio of polymer binder/casting solution, ratio of PVP to PES, ratio of Resin/polymer binder and casting temperature on ion exchange capacity (IEC) and permselectivity of the IEM	9
Hollow fiber Layer-by-Layer	NF	RSM	The dual-response surface analysis was utilized to identify the optimal process parameters with the objective of achieving a high membrane permeability and $MgSO_4$ rejection.	10
TFC-IP membranes	RO	RSM	Tested six preparation factors for polyamide layer formation to find optimum values for exceptional desalination performance	11
Chlorinated-Polypropylene membrane	Anion Exchange Membrane (AEM)	RSM	Membrane Design parameters were modelled using RSM to optimize IEC and permselectivity.	12

Table S2. Examples of input and output features used for ML models in polymeric membrane research.

Input features	Output features	Ref
Polymer type, polymer concentration, filler concentration, average filler size, solvent type, solvent concentration, contact angle	Solute rejection, pure water flux, flux recovery	13
Substrate type, amine monomer, amine monomer concentration, chloride monomer, chloride monomer concentration, water contact angle, surface roughness, nanomaterial type, nanomaterial size, nanomaterial loading.<br><br>Solvent and solute conditions include solvent type, molecular weight, viscosity, density, and molar volume, solute type, solute concentration, solute molecular weight, and solute charge.	Relative permeability and relative selectivity	14
Membrane properties: control water permeability, control salt pass rate, relative water contact angle, relative roughness,<br><br>Nanoparticle properties: shape, size, pore size, charge, solvent phase, bonding, and loading	Relative water permeability and relative salt pass rate	15
Pore radius, zeta potential, pressure, and concentration	Salt rejection (NaCl, Na_2SO_4, $MgCl_2$, and $MgSO_4$)	16
Total charge, water contact angle, membrane molecular weight cut-off (MWCO), compound size, compound log K_{ow}, hydraulic pressure, initial concentration of the compound, and measurement time	Micropollutant removal rate	17
Support, particle concentration, concentration of organic phase, operation pressure, CA, thin layer thickness, location of the NPs, post treatment temperature and duration	Permeate flux and foulant rejection	18
MOF Properties : MOF type, MOF Size, Bruner-Emmentt-Teller (BET) surface area, MOF pore diameter, loading of MOFs in the membrane<br><br>Polymer Properties: Target Coding and molecular descriptors<br><br>Operating Conditions: Temperature, Operating Pressure and proportion of CO_2 in mixed gas	Permeability and Gas Selectivity	19
Conductivity, IEC, Water Uptake, Swelling ratio, maximum elongation, maximum stress, membrane thickness, ether aryl contribution, alkyl chain contribution, sulfone contribution, spacer, extender, Alkali temperature, Alkali concentration	Conductivity retention (indicating stability of Anion Exchange Membranes)	20

Table S3. ML algorithms commonly used in polymeric membrane research.

Name	Category	Description	Ref
Linear/Multilinear Regression (LR/MLR)	Supervised	The basic ML algorithm used to form relationship between one or more independent variables with a dependent variable. The independent variable is represented as a weighted sum of the dependent variables.	14
Artificial Neural Networks (ANN)	Supervised	ANN consists of interconnected nodes, which are organized into layers (input, hidden, and output). Each specific connection in the layers has an associated weight (strength of the connection) and bias (additional parameters). These weights and biases are adjusted during model training to optimize the networks performance.	13,20,21
Random Forest (RF)	Supervised	RF is an ensemble learning approach which operates by constructing multiple decision trees during training. The final output given by the trees is determined by the consensus of multiple trees within a forest.	16,22,23
Extreme Gradient Boosting (XGBoost)	Supervised	XGBoost finds its roots in gradient boosting, a ML model wherein trees are built sequentially to minimize the errors made by previously trained trees.	24,25
Categorical Boosting (CatBoost)	Supervised	Similar to XGBoost, it is an implementation of gradient boosting. CatBoost was specifically designed to handle categorical data without any encoding.	24–26
Support vector machine (SVM)	Supervised	SVM aims to find a hyperplane in a high-dimensional space that best separates the data points between two classes.	14,23
K-means clustering	Unsupervised	It groups similar data points into clusters based on the similarities of their features.	27,28
Principal component analysis (PCA)	Unsupervised	PCA reduces dimensionality of the dataset while retaining the original information present in the data. The principal components are generated by finding the eigenvectors of the covariance matrix of the dataset.	29,30

References

(1) Kaushik, A.; Dhundhiyawala, M.; Dobariya, P.; Marvaniya, K.; Kushwaha, S.; Patel, K. Perm-Selective Ultrathin High Flux Microporous Polyaryl Nanofilm for Molecular Separation. *iScience* **2022**, *25* (6), 104441. https://doi.org/10.1016/j.isci.2022.104441.

(2) Nazarov, I. V.; Khrychikova, A. P.; Medentseva, E. I.; Bermesheva, E. V.; Borisov, I. L.; Yushkin, A. A.; Volkov, A. V.; Wozniak, A. I.; Petukhov, D. I.; Topchiy, M. A.; Asachenko, A. F.; Ren, X.-K.; Bermeshev, M. V. CO2-Selective Vinyl-Addition Polymers from Nadimides: Synthesis and Performance for Membrane Gas Separation. *J. Membr. Sci.* **2023**, *677*, 121624. https://doi.org/10.1016/j.memsci.2023.121624.

(3) Burts, K. S.; Plisko, T. V.; Bildyukevich, A. V.; Li, G.; Kujawa, J.; Kujawski, W. Development of Dynamic PVA/PAN Membranes for Pervaporation: Correlation between Kinetics of Gel Layer Formation, Preparation Conditions, and Separation Performance. *Chem. Eng. Res. Des.* **2022**, *182*, 544–557. https://doi.org/10.1016/j.cherd.2022.04.016.

(4) Peng, Q.; Lu, Y.; Fang, W.; Zhu, Y.; Jin, J. Attenuated Thermal-Regulated Interfacial Polymerization towards Polyamide Nanofiltration Membrane with Unprecedentedly Enhanced Performance. *Chem. Eng. J.* **2023**, *471*, 144706. https://doi.org/10.1016/j.cej.2023.144706.

(5) Ghazali, A. A.; Roshan, R. K.; Rahman, S. A.; Wahab, M. S. A.; Hazleen, N. S.; Mandayar, N. 23 Fractional Factorial Design for Polymer Based Thin Film Composite (TFC) Membrane Synthesis for CO2/CH4 Separation. *IOP Conf. Ser. Mater. Sci. Eng.* **2019**, *702* (1), 012049. https://doi.org/10.1088/1757-899X/702/1/012049.

(6) Berradi, M.; Berradi, O.; Chellouli, M.; Hsissou, R.; El Bouchti, M.; El Gouri, M.; Sallek, B.; El Bachiri, A.; El Harfi, A. Optimization of the Synthesis of Ultrafiltration Asymmetric Membranes Based on Organic Polymers. *Results Eng.* **2020**, *6*, 100116. https://doi.org/10.1016/j.rineng.2020.100116.

(7) Idris, A.; Ismail, A. F.; Noordin, M. Y.; Shilton, S. J. Optimization of Cellulose Acetate Hollow Fiber Reverse Osmosis Membrane Production Using Taguchi Method. *J. Membr. Sci.* **2002**, *205* (1), 223–237. https://doi.org/10.1016/S0376-7388(02)00116-3.

(8) Amirilargani, M.; Sadrzadeh, M.; Mohammadi, T. Synthesis and Characterization of Polyethersulfone Membranes. *J. Polym. Res.* **2010**, *17* (3), 363–377. https://doi.org/10.1007/s10965-009-9323-6.

(9) Mofrad, A. E.; Moheb, A.; Masigol, M.; Sadeghi, M.; Radmanesh, F. An Investigation into Electrochemical Properties of Poly(Ether Sulfone)/Poly(Vinyl Pyrrolidone) Heterogeneous Cation-Exchange Membranes by Using Design of Experiment Method. *J. Colloid Interface Sci.* **2018**, *532*, 546–556. https://doi.org/10.1016/j.jcis.2018.08.026.

(10) Wang, M.; Liu, C.; Fan, M.; Liu, M.; Shen, S. Optimization of Nanofiltration Hollow Fiber Membrane Fabrication Process Based on Response Surface Method. *Membranes* **2022**, *12* (4), 374. https://doi.org/10.3390/membranes12040374.

(11) Vatanpour, V.; Sheydaei, M.; Esmaeili, M. Box-Behnken Design as a Systematic Approach to Inspect Correlation between Synthesis Conditions and Desalination

Performance of TFC RO Membranes. *Desalination* **2017**, *420*, 1–11. https://doi.org/10.1016/j.desal.2017.06.022.
(12) Kikhavani, T.; Ashrafizadeh, S. N.; Van der Bruggen, B. Identification of Optimum Synthesis Conditions for a Novel Anion Exchange Membrane by Response Surface Methodology. *J. Appl. Polym. Sci.* **2014**, *131* (3). https://doi.org/10.1002/app.39888.
(13) Fetanat, M.; Keshtiara, M.; Low, Z.-X.; Keyikoglu, R.; Khataee, A.; Orooji, Y.; Chen, V.; Leslie, G.; Razmjou, A. Machine Learning for Advanced Design of Nanocomposite Ultrafiltration Membranes. *Ind. Eng. Chem. Res.* **2021**, *60* (14), 5236–5250. https://doi.org/10.1021/acs.iecr.0c05446.
(14) Wang, C.; Wang, L.; Soo, A.; Bansidhar Pathak, N.; Kyong Shon, H. Machine Learning Based Prediction and Optimization of Thin Film Nanocomposite Membranes for Organic Solvent Nanofiltration. *Sep. Purif. Technol.* **2023**, *304*, 122328. https://doi.org/10.1016/j.seppur.2022.122328.
(15) Yeo, C. S. H.; Xie, Q.; Wang, X.; Zhang, S. Understanding and Optimization of Thin Film Nanocomposite Membranes for Reverse Osmosis with Machine Learning. *J. Membr. Sci.* **2020**, *606*, 118135. https://doi.org/10.1016/j.memsci.2020.118135.
(16) Ma, X.; Lu, D.; Lu, J.; Qian, Y.; Zhang, S.; Yao, Z.; Liang, L.; Sun, Z.; Zhang, L. Revealing Key Structural and Operating Features on Water/Salts Selectivity of Polyamide Nanofiltration Membranes by Ensemble Machine Learning. *Desalination* **2023**, *548*, 116293. https://doi.org/10.1016/j.desal.2022.116293.
(17) Jeong, N.; Chung, T.; Tong, T. Predicting Micropollutant Removal by Reverse Osmosis and Nanofiltration Membranes: Is Machine Learning Viable? *Environ. Sci. Technol.* **2021**, *55* (16), 11348–11359. https://doi.org/10.1021/acs.est.1c04041.
(18) Fetanat, M.; Keshtiara, M.; Keyikoglu, R.; Khataee, A.; Daiyan, R.; Razmjou, A. Machine Learning for Design of Thin-Film Nanocomposite Membranes. *Sep. Purif. Technol.* **2021**, *270*, 118383. https://doi.org/10.1016/j.seppur.2021.118383.
(19) Yao, L.; Zhang, Z.; Li, Y.; Zhuo, J.; Chen, Z.; Lin, Z.; Liu, H.; Yao, Z. Precise Prediction of CO2 Separation Performance of Metal–Organic Framework Mixed Matrix Membranes Based on Feature Selection and Machine Learning. *Sep. Purif. Technol.* **2024**, *349*, 127894. https://doi.org/10.1016/j.seppur.2024.127894.
(20) Zou, X.; Pan, J.; Sun, Z.; Wang, B.; Jin, Z.; Xu, G.; Yan, F. Machine Learning Analysis and Prediction Models of Alkaline Anion Exchange Membranes for Fuel Cells. *Energy Environ. Sci.* **2021**, *14* (7), 3965–3975. https://doi.org/10.1039/D1EE01170G.
(21) Hasnaoui, H.; Krea, M.; Roizard, D. Neural Networks for the Prediction of Polymer Permeability to Gases. *J. Membr. Sci.* **2017**, *541*, 541–549. https://doi.org/10.1016/j.memsci.2017.07.031.
(22) Sawada, S.; Sakamoto, Y.; Funatsu, K.; Maekawa, Y. Toward the Design of Graft-Type Proton Exchange Membranes with High Proton Conductivity and Low Water Uptake: A Machine Learning Study. *J. Membr. Sci.* **2024**, *692*, 122169. https://doi.org/10.1016/j.memsci.2023.122169.
(23) Zhang, Q.; Yuan, Y.; Zhang, J.; Fang, P.; Pan, J.; Zhang, H.; Zhou, T.; Yu, Q.; Zou, X.; Sun, Z.; Yan, F. Machine Learning-Aided Design of Highly Conductive Anion Exchange Membranes for Fuel Cells and Water Electrolyzers. *Adv. Mater.* **2024**, *36* (36), 2404981. https://doi.org/10.1002/adma.202404981.

(24) Gao, H.; Zhong, S.; Dangayach, R.; Chen, Y. Understanding and Designing a High-Performance Ultrafiltration Membrane Using Machine Learning. *Environ. Sci. Technol.* **2023**. https://doi.org/10.1021/acs.est.2c05404.
(25) Yuyama, S.; Kaneko, H. Simultaneous Design of Gas Separation Membranes and Schemes through Combined Process and Materials Informatics. *Ind. Eng. Chem. Res.* **2023**, *62* (44), 18541–18551. https://doi.org/10.1021/acs.iecr.3c02444.
(26) Ding, R.; Yin, W.; Cheng, G.; Chen, Y.; Wang, J.; Wang, X.; Han, M.; Zhang, T.; Cao, Y.; Zhao, H.; Wang, S.; Li, J.; Liu, J. Effectively Increasing Pt Utilization Efficiency of the Membrane Electrode Assembly in Proton Exchange Membrane Fuel Cells through Multiparameter Optimization Guided by Machine Learning. *ACS Appl. Mater. Interfaces* **2022**, *14* (6), 8010–8024. https://doi.org/10.1021/acsami.1c23221.
(27) Yin, H.; Xu, M.; Luo, Z.; Bi, X.; Li, J.; Zhang, S.; Wang, X. Machine Learning for Membrane Design and Discovery. *Green Energy Environ.* **2024**, *9* (1), 54–70. https://doi.org/10.1016/j.gee.2022.12.001.
(28) Dansawad, P.; Li, Y.; Li, Y.; Zhang, J.; You, S.; Li, W.; Yi, S. Machine Learning toward Improving the Performance of Membrane-Based Wastewater Treatment: A Review. *Adv. Membr.* **2023**, *3*, 100072. https://doi.org/10.1016/j.advmem.2023.100072.
(29) Wang, M.; Xu, Q.; Tang, H.; Jiang, J. Machine Learning-Enabled Prediction and High-Throughput Screening of Polymer Membranes for Pervaporation Separation. *ACS Appl. Mater. Interfaces* **2022**, *14* (6), 8427–8436. https://doi.org/10.1021/acsami.1c22886.
(30) Phua, Y. K.; Terasoba, N.; Tanaka, M.; Fujigaya, T.; Kato, K. Unsupervised Machine Learning-Derived Anion-Exchange Membrane Polymers Map: A Guideline for Polymers Exploration and Design. *ChemElectroChem* **2024**, *11* (14), e202400252. https://doi.org/10.1002/celc.202400252.
